# Effect of long-term combined application of organic and inorganic fertilizers on soil nematode communities within aggregates

**DOI:** 10.1038/srep31118

**Published:** 2016-08-09

**Authors:** Zhiyong Zhang, Xiaoke Zhang, Md. Mahamood, Shuiqing Zhang, Shaomin Huang, Wenju Liang

**Affiliations:** 1 Liaoning Key Laboratory of Soil Environmental Quality and Agro-product Safety, Institute of Applied Ecology, Chinese Academy of Sciences, Shenyang 110016, China; 2 College of Land and Environment, Shenyang Agricultural University, Shenyang 110161, China; 3 Institute of Plant Nutrition and Environmental Resources Science, Henan Academy of Agricultural Sciences, Zhengzhou 450002, China

## Abstract

A long-term fertilization experiment was conducted to examine the effects of different fertilization practices on nematode community composition within aggregates in a wheat-maize rotation system. The study was a randomized complete block design with three replicates. The experiment involved the following four treatments: no fertilizer, inorganic N, P and K fertilizer (NPK), NPK plus manure (NPKM) and NPK plus maize straw (NPKS). Soil samples were taken at 0–20 cm depth during the wheat harvest stage. Based on our results, NPKS contributed to soil aggregation and moisture retention, with a positive effect on soil total nitrogen accumulation, particularly within small macroaggregates (0.25–1 mm) and microaggregates (<0.25 mm). The C/N ratio was correlated to the distribution of the soil nematode community. Both manure application and straw incorporation increased the nematode functional metabolic footprints within all aggregates. Additionally, the functional metabolic footprints decreased with a decline in aggregate size. The accumulation of total nitrogen within <1 mm aggregates under NPKS might play a key role in maintaining the survival of soil nematodes. In our study, both crop straw incorporation and inorganic fertilizer application effectively improved soil physicochemical properties and were also beneficial for nematode survival within small aggregate size fractions.

In modern agriculture, to attain high crop yields and satisfy the demand of an increasing population, a significant amount of inorganic fertilizers is applied to croplands^1^. However, long-term inputs of inorganic fertilizers are disadvantageous to develop long-term sustainability in agroecosystems^2^. Organic inputs are widely accepted as one of the sustainable agricultural practices that improve soil fertility and soil biological properties^3^,^4^,^5^. Considering the requirement for both crop yields and sustainable agroecosystems, the combined application of inorganic fertilizers with organic materials is regarded as a reasonable and effective approach to achieve both goals^6^. Chivenge *et al.*^7^ concluded that the reasonable application of inorganic fertilizers with organic resources can increase soil fertility and crop yields.

Soil aggregates are formed from the combination of soil organic matter and soil mineral particles (sand, silt and clay)^8^. Size and stability of aggregates influence the composition and activity of soil biota^9^,^10^,^11^. Among the soil fauna, soil nematodes are abundant soil invertebrates occupying central position of soil food webs and have a pronounced influence on soil ecological processes^12^,^13^. The distribution of nematodes in soils is dependent on feeding habits, body size and the availability of resources^14^,^15^. Zhang *et al.*^16^ found that soil nematode abundance decreased with decreasing aggregate size. However, according to Briar *et al.*^15^, numbers of nematodes are higher within microaggregates (<250 μm) than within small macroaggregates (250–1000 μm). Soil aggregates of different size fractions might offer spatially heterogeneous habitats for soil nematodes. Additionally, the relationship between microbial and nematode communities also determines nematode distribution^15^,^16^. Therefore, to understand the functions of soil biota in ecosystems, determination of their changes within microhabitats is essential, particularly within different aggregate size fractions^17^.

Previously, many researchers focused attention on the effects of tillage practices on the distribution of nematode assemblage within soil aggregate fractions^15^,^16^. Tillage practices influence soil nematodes directly through physical disruption of soil microhabitats. Some studies investigated the responses of soil nematodes to different types of fertilization management, but few works reported the distribution of soil nematodes within soil aggregate fractions under fertilization practices. Although Jiang *et al.*^18^ determined the nematode community distribution within aggregates under different application rates of manure, the variation in nutrient status and in soil nematode communities within aggregates following the combined application of inorganic fertilizers with different organic materials remains unclear. Therefore, to assess the effect of fertilization practices on the soil micro-food web in micro-scale environments, the variation in soil nematode communities within different aggregate size fractions must be determined.

The objectives of this study were to investigate the effects of the combined application of organic and inorganic fertilizers on soil aggregate stability, to analyse the variation in soil nematode communities within aggregates under different fertilization practices, and to identify the relationship between soil physicochemical properties and the distribution of nematode communities in different fertilization treatments. We hypothesized that compared with manure application, straw incorporation might be more effective in increasing soil nutrients within aggregates, and that nematode metabolic footprints within aggregate fractions changed with the type of fertilization.

## Results

### Soil aggregation and physicochemical properties within aggregates

The soil mean weight diameter was greater in NPKS than in other treatments (Table 1, P < 0.05). Compared with NPK, the proportions of >2 mm and 1–2 mm aggregates of NPKS were increased 13.30% and 18.84%, respectively. In both NPKM and NPKS, the proportions of <0.25 mm aggregates decreased significantly in comparison with CK and NPK (P < 0.05). Two-way Analysis of Variance (ANOVA) showed significant influences of fertilization, aggregate size and their interaction on soil physicochemical properties (Table 2, P < 0.05). Higher soil moisture was found in NPKS than in the other treatments, except for <0.25 mm aggregates (Table 2, P < 0.05). Both soil organic carbon and total nitrogen were strongly affected by fertilization, with greater contents observed in NPKM and NPKS within all aggregates (Table 2, P < 0.05). However, the relation with C/N ratios was different from that observed for soil organic carbon and total nitrogen, with higher C/N ratios observed in CK than those in NPKM and NPKS (Table 2, P < 0.05). In general, the effects of aggregate size on soil physicochemical properties were significant, and soil moisture, soil organic carbon, total nitrogen and C/N ratios were lower within <0.25 mm aggregates than within >0.25 mm aggregates (Table 2, P < 0.05). In comparison with CK, soil pH was lower in the other three fertilization treatments (P < 0.05). Moreover, lower pH values were found within <0.25 mm aggregates than within >0.25 mm aggregates (P < 0.05).

The accumulation of soil organic carbon and total nitrogen were higher within >0.25 mm aggregate size fractions than within <0.25 mm aggregate fractions (Table 2, P < 0.05). Soil aggregate size and the interaction of fertilization and aggregate size also significantly affected the enrichment factors of soil organic carbon and total nitrogen within aggregates (E_SOC_ and E_TN_, respectively; Table 2, P < 0.05). Additionally, higher E_TN_ were observed in NPKS than in the other three treatments within 0.25–1 mm and <0.25 mm aggregate fractions (Table 2, P < 0.05).

### Soil microbial biomass within aggregates in different fertilization treatments

Soil microbial biomass was strongly affected by fertilization and aggregate size (Table 3). In comparison with CK and NPK, in both NPKM and NPKS, total microbial biomass increased within all aggregates (P < 0.05). Additionally, bacterial biomass was higher in NPKM and NPKS than in CK and NPK within all aggregates (P < 0.05). Except for the 0.25–1 mm fraction, greater fungal biomass was found in NPKM and NPKS than in CK (Table 3, P < 0.05). Furthermore, relatively lower microbial biomass was found within 0.25–1 mm than within 1–2 mm aggregates (P < 0.05).

### Nematode community distribution and metabolic footprints within different aggregate size fractions

**Figure 1:**
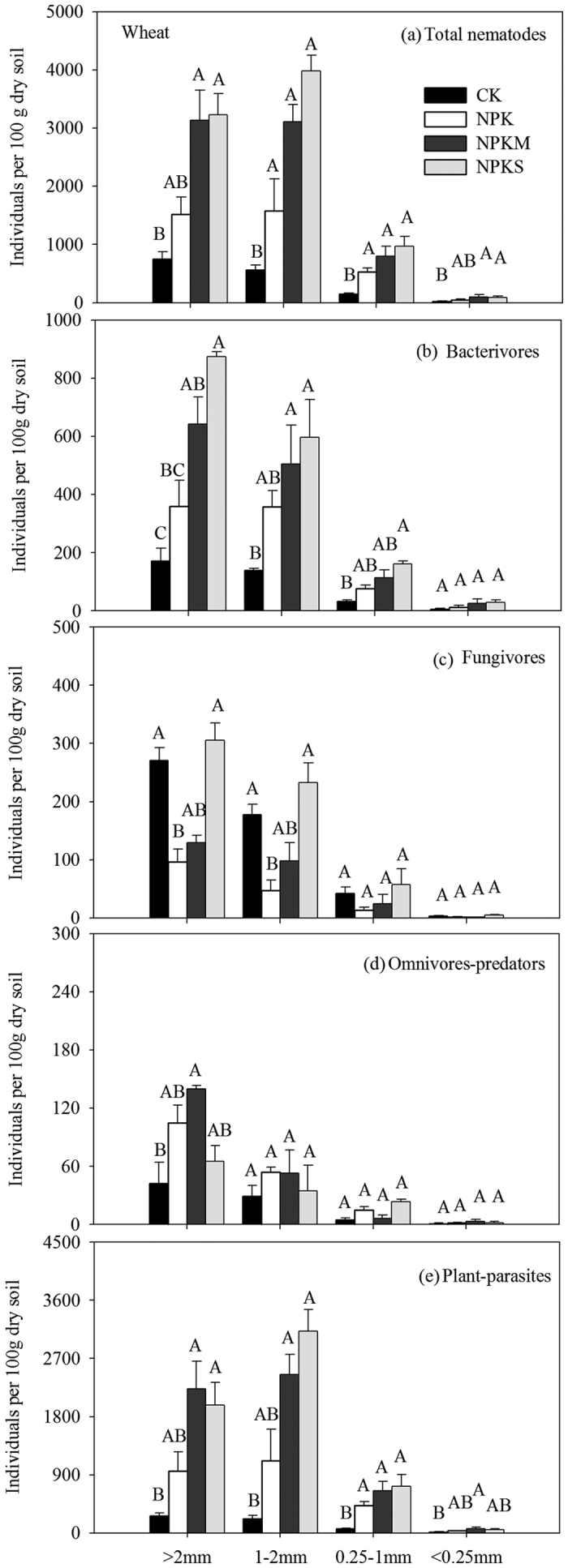
Abundances of total nematodes and different trophic groups within aggregates under different fertilization treatments. Bars indicate standard error (n = 3). Different capital letters (A,B etc.) show significant differences among different treatments for each aggregate size, respectively, as determined by Tukey’s honestly significant difference test, P < 0.05.

Twenty-two nematode genera were identified in the wheat season (Supplementary Table S1). The dominant nematode genus was *Geocenamus*, with a relative abundance >10% within all aggregates among the four fertilization treatments. Fertilization practices strongly affected soil nematode genera. For example, fungivores such as *Filenchus*, *Diphtherophora* and *Tylencholaimus* were relatively more abundant in CK than in the other three treatments (P < 0.05). Additionally, in this study, *Prismatolaimus*, *Eudorylaimus* and *Basiria* were observed only within >0.25 mm aggregate size fractions (Supplementary Table S1). Fertilization practices also strongly affected the total nematode abundance and that of different trophic groups (Fig. 1, P < 0.05). The abundances of total nematodes, bacterivores and plant-parasites were higher in NPKM and NPKS than in CK, and fungivores were more abundant in CK and NPKS than in NPK within >2 mm and 1–2 mm aggregates (Fig. 1a–c,e, P < 0.05). In comparison with CK, only in NPKM, the abundance of omnivores-predators increased within >2 mm aggregate fraction (Fig. 1d, P < 0.05). Within 0.25–1 mm aggregates, higher abundances of total nematodes, bacterivores and plant-parasites were observed in NPKS than in CK (P < 0.05). Additionally, in NPKM, abundances of total nematodes and plant-parasites increased significantly in comparison with CK within <0.25 mm aggregates (P < 0.05). The soil nematode community was also sensitive to the aggregate size fraction, and abundances of total nematodes and the four trophic groups were higher within >2 mm, 1–2 mm and 0.25–1 mm than within <0.25 mm aggregates (Fig. 1, P < 0.05).

**Figure 2:**
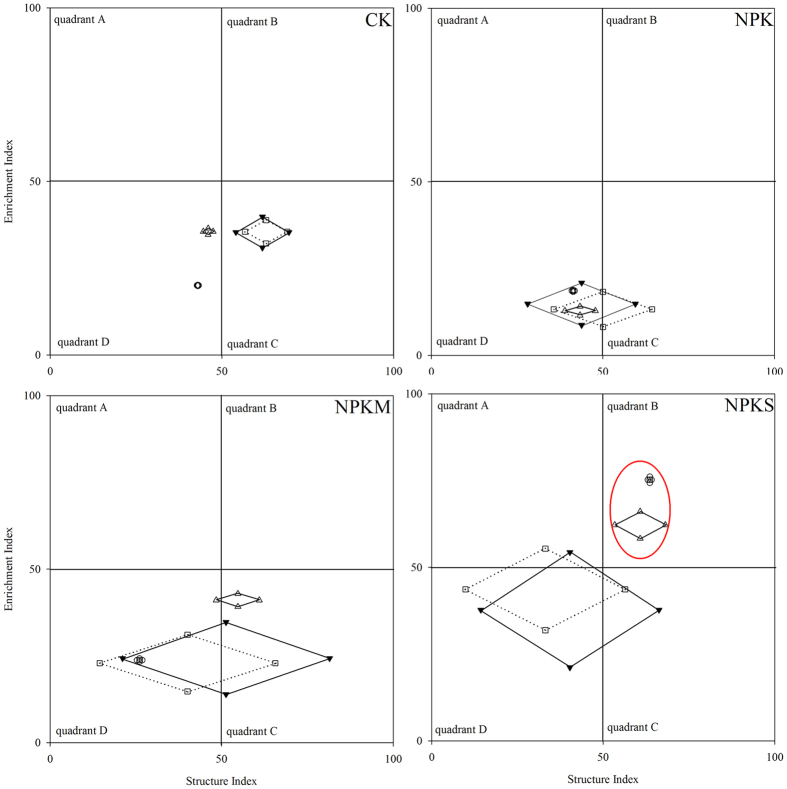
Functional metabolic footprint of nematodes subjected to fertilization in the wheat season (▾, ◻, ∆, ⚪ represent >2 mm, 1–2 mm, 0.25–1 mm and <0.25 mm, respectively). The vertical axis and horizontal axis of each footprint represent the enrichment footprint and structure footprint, respectively. The functional metabolic footprint is described by the sequentially joining points: (SI-0.5Fs/k, EI); (SI, EI + 0.5Fe/k); (SI + 0.5Fs/k, EI); (SI, EI-0.5Fe/k). Fs and Fe represent the structure footprint and enrichment footprint, respectively. EI and SI represent enrichment index and structure index, respectively, and the k value is 3. The nematode functional metabolic footprint is the total area of the two functional (enrichment and structure) footprints^46^,^56^.

Fertilization had significant effects on nematode enrichment and structure indices (Table 4, P < 0.05). Except for <0.25 mm aggregates, the enrichment index was lower in NPK than in the other three treatments (P < 0.05). Within >2 mm and 1–2 mm aggregates, the structure index was higher in CK in comparison with NPKS (P < 0.05). Additionally, only the structure index was strongly influenced by aggregate size, with relatively lower values found within <0.25 mm aggregates. Fertilization practices and aggregate size significantly influenced the functional metabolic footprints of soil nematodes (Fig. 2, P < 0.05). Within all aggregates, greater functional metabolic footprints were found in NPKM and NPKS than in NPK and CK (P < 0.05). Additionally, the values of functional metabolic footprints significantly decreased with decreasing aggregate size (Fig. 2, P < 0.05). Except for CK, the functional metabolic footprints within >2 mm and 1–2 mm aggregates in the other three fertilization treatments primarily moved from quadrant C to quadrant D. Except for 0.25–1 mm and <0.25 mm aggregate fractions for NPKS in quadrant B, the other aggregates under different fertilization treatments were all clearly located in the area in which the enrichment indexes were <50 (i.e., quadrants C and D; Fig. 2).

### Relationships between nematodes and soil physicochemical properties within aggregates

**Figure 3:**
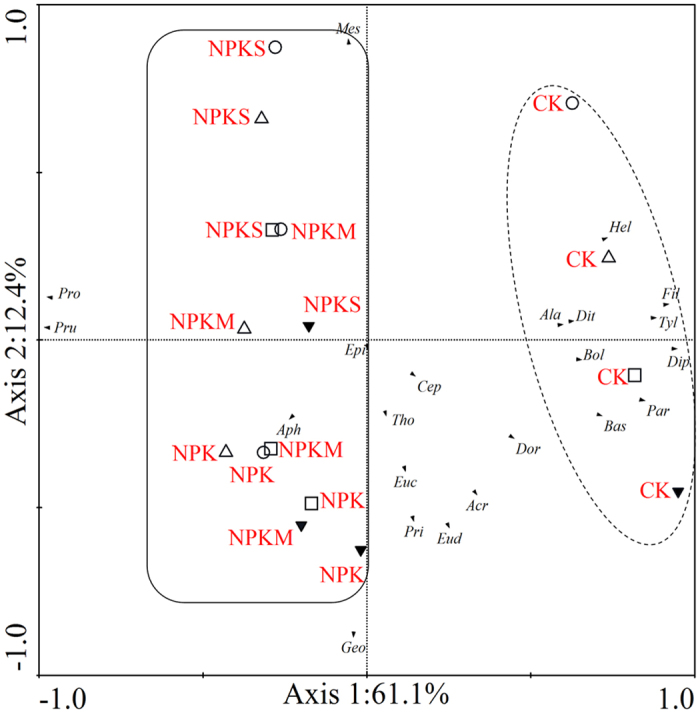
Principle component analysis of nematode genera composition within various aggregate fractions under different fertilization treatments. ▾, ◻, ∆, ⚪ represent >2 mm, 1–2 mm, 0.25–1 mm and <0.25 mm, respectively. *Mesorhabditis, Mes; Cephalobus, Cep; Eucephalobus, Euc; Acrobeloides, Acr; Prismatolaimus, Pri; Alaimus, Ala; Filenchus, Fil; Ditylenchus, Dit; Paraphelenchus, Par; Aphelenchoides, Aph; Diphtherophora, Dip; Tylencholaimus, Tyl; Thonus, Tho; Eudorylaimus, Eud; Epidorylaimus, Epi; Dorydorella, Dor; Boleodorus, Bol; Basiria, Bas; Geocenamus, Geo; Helicotylenchus, Hel; Pratylenchus, Pru; Pratylenchoides, Pro*.

**Figure 4:**
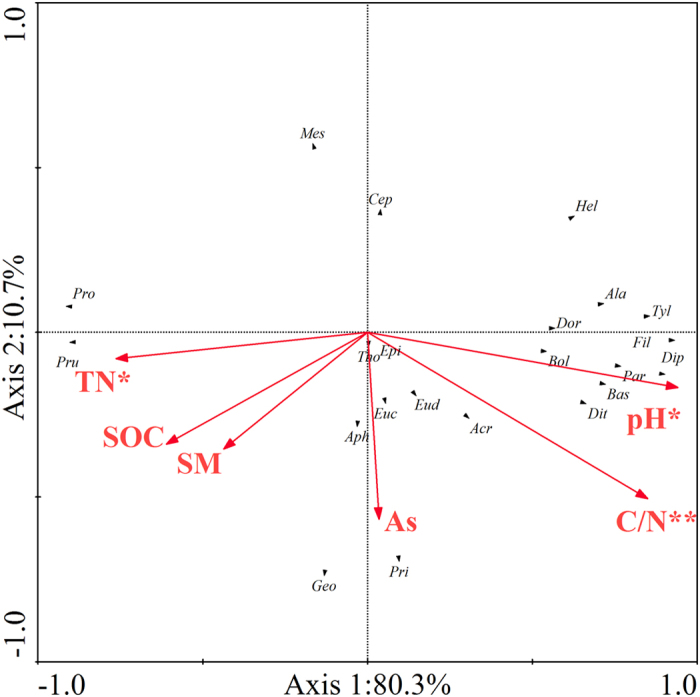
Redundancy analysis of the relationship between nematode genera and soil physiochemical properties in the wheat season. SOC, soil organic carbon; TN, total nitrogen; SM, soil moisture; As, aggregate size; C/N, C/N ratio; **P < 0.01; *P < 0.05. *Mesorhabditis, Mes; Cephalobus, Cep; Eucephalobus, Euc; Acrobeloides, Acr; Prismatolaimus, Pri; Alaimus, Ala; Filenchus, Fil; Ditylenchus, Dit; Paraphelenchus, Par; Aphelenchoides, Aph; Diphtherophora, Dip; Tylencholaimus, Tyl; Thonus, Tho; Eudorylaimus, Eud; Epidorylaimus, Epi; Dorydorella, Dor; Boleodorus, Bol; Basiria, Bas; Geocenamus, Geo; Helicotylenchus, Hel; Pratylenchus, Pru; Pratylenchoides, Pro.*

Principle component analysis (Fig. 3) showed that nematode community compositions in CK and in NPK, NPKM and NPKS were significantly distinguished by the first canonical axis, which explained 61.1% of the total variation. However, in general, the second canonical axis (12.4%) did not distinguish clearly the nematode communities within different aggregate fractions. Based on the different distributions of soil nematodes under different fertilization practices, redundancy analysis (Fig. 4) was performed to analyse the relationship between nematodes and soil physicochemical properties. Among the soil physicochemical properties, C/N ratio was the primary driving factor that influenced soil nematode communities (Fig. 4, P < 0.01). Additionally, soil nematode communities were affected by soil pH and total nitrogen (P < 0.05).

## Discussion

In our study, the long-term application of crop straw into soils significantly affected soil physicochemical properties such as the mean weight diameter, soil moisture, soil organic carbon and total nitrogen across all aggregate sizes (Tables 1 and 2). In comparison with treatments without crop residue application, straw incorporation improves soil hydrological properties^19^, such as water-holding capacity^20^, which results in higher water content in soils^21^. Additionally, the application of straw increased carbon resources, which contributed to soil organic carbon accumulation. Furthermore, lignin and cellulose derived from maize straw forms organic cementing material to increase soil aggregate stability^22^,^23^. Although both manure application and straw incorporation increased the contents of soil organic carbon and total nitrogen, C/N ratios were relatively lower in the two organic fertilization treatments (Table 2). In general, during the decomposition of manure, C is lost faster than N, which leads to a decrease in C/N ratio in manure treatments^24^. In the present study, in contrast to the other treatments, enrichment factors for total nitrogen were higher within 0.25–1 mm and <0.25 mm aggregates in NPKS (Table 2). Therefore, straw incorporation might have an important role in contributing to total nitrogen storage, particularly within <1 mm aggregates.

The accumulation of soil organic carbon functional groups within aggregates is dependent on soil aggregate size rather than nutrient amendments^25^. In this study, soil organic carbon contents were higher within macroaggregates (>0.25 mm aggregates) than within microaggregates and silt and clay fractions (<0.25 mm aggregates). This observation was consistent with the studies of Xie *et al.*^26^ and Lichter *et al.*^27^, who demonstrated that both manure application and residue incorporation increase soil organic carbon in macroaggregates. Tripathi *et al.*^28^ concluded that the incorporation of organic fertilizers causes the decomposition of organic matter and then roots, hyphae and polysaccharides bind mineral particles into microaggregates, which then contribute to C-enrichment in macroaggregates.

In this study, both NPKM and NPKS had positive effects on total nematode abundances within aggregates. External organic inputs of manure and crop straw increase energy availability for soil microbes and thereby increase microbial activity and biomass^29^. In our study, significantly positive relationships between total nematodes and soil microbial biomass were found in the manure and straw incorporation treatments (P < 0.05). Therefore, the increase in microbial biomass likely led to the higher total nematode abundance within all aggregates in NPKS and NPKM treatments. Within all aggregates, the abundance of plant-parasites clearly increased with manure application compared with CK. Based on the manure application rate, our results were partially consistent with the observations of Jiang *et al.*^18^, who found that low-rate application of manure (150 kg N ha^−1^ y^−1^) leads to increases in numbers of plant-parasites but that high-rate application (600 kg N ha^−1^ y^−1^) may have negative effects. Long-term fertilization, particularly amendments with manure, increases root density and also the development of external mycelia of arbuscular mycorrhizal fungi^30^,^31^. Fungal hyphae and plant roots that remain in soils bind microaggregates into macroaggregates, which may offer more feeding spaces and resources for plant-parasites^23^. The data for *Basiria* belonged to plant-parasites partially confirmed this hypothesis (Supplementary Table S1). In this study, dry sieving method for aggregate fractionation was performed. The dry-sieving procedure was adopted in order to avoid the loss of hydrotropic nematodes^32^. However, the wet-sieving method could increase the loss of microbial biomass living on the surface of aggregate fractions^18^. It must be noted that there is still limit for dry sieving method due to the moisture control during the separation and the power of separating soil blocks into fragments by hand before the mechanical sieving.

Analyses of nematode fauna using enrichment and structure indices provide information on the status of soil food webs^33^. Moreover, the nematode enrichment index is a reflection of the response of primary decomposers such as bacterivores (cp 1) and fungivores (cp 2) to resources entering the soil food web^33^. In this study, the lowest enrichment index was in the NPK treatment, which was indication of the relatively small labile nutrient pool in the inorganic fertilization treatments, and with limited nutrients the development of the soil food web might be restricted^34^. The structure index is primarily dependent on populations of omnivores-predators, which are sensitive to soil disturbance and require long recovery times^33^. In comparison with NPKS, greater values of structure index within >1 mm aggregates in CK indicated that the soil food web was a relatively complex community with few disturbances within larger aggregates^34^. Nematode metabolic footprints provide information on the responses of nematode assemblages to resources but are also an indication of the functions and services offered by nematodes^35^. In the present study, both manure application and straw incorporation increased soil nematode functional metabolic footprints within all aggregates (Fig. 2). A larger nematode functional footprint indicates that more C flows into the soil nematode community, which is used for nematode production^36^. Consequently, based on our results, organic inputs likely had a positive effect on carbon accumulation in the soil nematode community. Additionally, for the nematode functional metabolic footprint across all fertilization treatments, the ranking was as follows: >2 mm aggregate >1–2 mm aggregate >0.25–1 mm aggregate >aggregate <0.25 mm (<0.25 mm aggregate). Two possibilities might explain the decline in the nematode functional metabolic footprint with the decrease in aggregate size. First, the relatively low microbial biomass within the smaller aggregates did not support the development of the nematode community. Additionally, because of the large body size of some nematodes, access to the small aggregate fraction may be restricted, leading to a decrease in nematode abundance. For example, in this study, both *Prismatolaimus* and *Eudorylaimus* did not appear within small aggregate size fractions. Typically, nematodes such as *Prismatolaimus* and *Eudorylaimus* with high cp values have relatively large body sizes, and therefore, the space within smaller aggregate sizes may limit access. In a nematode faunal analysis, Ferris *et al.*^33^ concluded that the soil food web is structured when in quadrant C (EI < 50, SI > 50); however, when in quadrant D (EI < 50, SI < 50), the soil food web is likely degraded. In this research, the tendency of nematode functional metabolic footprints within >1 mm aggregates in NPK, NPKM and NPKS to move from quadrant C to quadrant D indicated that the soil disturbance caused by fertilization practices likely strongly affected the soil food web, particularly within larger aggregates. Notably, the nematode functional metabolic footprints of 0.25–1 mm and <0.25 mm aggregate fractions for NPKS, with relatively greater enrichment and structure indices, were located in quadrant B and were different from the identical aggregate fractions in the other fertilization treatments (Fig. 2). This result was consistent with the enrichment factor for total nitrogen within aggregates of this study (Table 2). Nitrogen is a factor that limits primary production and with adequate N, the soil food web is maintained^33^. Therefore, the incorporation of crop straw might have a positive effect on soil nematode fauna, particularly within <1 mm aggregates, due to contributions to N accumulation in the smaller aggregates.

In the present study, among the environmental factors, the C/N ratio had the strongest effect on the soil nematode community within aggregates (Fig. 4), with most soil nematode genera positively correlated with soil C/N ratio (Supplementary Table S2). The variation of C/N ratios within aggregates is a reflection of the quality of organic materials within aggregate fractions^37^. Furthermore, aggregates with higher C/N ratios indicate that the soil organic carbon is correspondingly fresh or little decomposed by microorganisms. However, low C/N ratios within aggregates suggest that the soil organic carbon is relatively aged and microbially derived^38^. Therefore, soil organic matter of differing quality within aggregates might lead to differences in availability of nutrients, which might indirectly affect soil nematodes.

In some studies, soil pH is the driving factor for changes in soil food webs^39^,^40^. In our research, soil pH also had a significant influence on soil nematode communities (Fig. 4), particularly for fungivores and plant-parasites (Supplementary Table S2). The decline of fungivores in the N input treatments was similar to that reported by Li *et al.*^41^, who found that the abundance of fungivores decreased in N addition plots. Following N addition, soil acidification is a key factor that inhibits soil nematodes^42^. In this study, both *Pratylenchus* and *Pratylenchoides* belonging to plant-parasites were negatively correlated with soil pH (Supplementary Tables S1 and S2). Lal and Jauhari^43^ observed that a change in soil pH from neutral to high suppressed the abundance of *Pratylenchus*. In this study, the application of inorganic or organic fertilizers led to a decline in soil pH but the soil remained slightly alkaline. Therefore, we inferred that the increasing abundance of *Pratylenchus* and *Pratylenchoides* might be related to the weakened suppressive effects induced by the decrease in soil pH.

In conclusion, both crop straw incorporation and inorganic fertilizer application contributed to soil aggregation, moisture retention and soil total nitrogen accumulation, particularly within <1 mm aggregates. The accumulation of total nitrogen within <1 mm aggregates under straw incorporation treatment might play an important role in maintaining the survival of soil nematodes. Soil C/N ratio exhibited a strong correlation with the nematode abundance in the present study. Organic inputs such as manure application or straw incorporation increased the nematode functional metabolic footprints within all aggregates. In addition, the functional metabolic footprints decreased with decreasing aggregate size. Understanding the distribution of soil nematode community within aggregate fractions not only reflected the nutrient status of aggregates but also clarified the effect of fertilization practices on the soil micro-food web within the micro-scale environments.

## Methods

### Experimental site and design

The study was conducted at the long-term fertilization experimental site of the Henan Academy of Agricultural Sciences in Yuanyang County, Henan Province, China (35°00′28′′ N, 113°41′48′′ E). The climate of the region is temperate monsoonal. The average annual precipitation is 646 mm, the mean annual temperature is 14.8 °C and the frost-free period is 224 d at this site. The soil is classified as Calcaric Cambisol (FAO classification) with initial soil basic properties as follow: soil pH (water: soil = 1:1) 8.3, organic matter 11 g kg^−1^, total N 0.6 g kg^−1^, total P 0.6 g kg^−1^ and total K 25.4 g kg^−1 ^44.

The experiment was established in 1990. A winter wheat (*Triticum aestivum* L.)-summer maize (*Zea mays* L.) rotation system was applied in all treatments each year. Winter wheat was sown in early October and harvested in early June. Summer maize was sown in early June and harvested in mid-September.

The experiment was a randomized block design with three replicates. Each experimental plot was 51 m^2^ (8.5 m × 6 m). The following four treatments were applied: (1) CK (no fertilizer), (2) NPK (N, urea; P, superphosphate; and K, potassium sulphate), (3) NPKM (NPK plus cattle manure compost) and (4) NPKS (NPK plus maize straw). Except for CK, the total N applied was equal in the three fertilization treatments. The amount of manure and crop straw applied was calculated based on the concentration of N in manure or crop straw, with the ratio of 7:3 maintained for manure-N or straw-N to mineral N. The average nutrient content in cattle manure was 13.1 g kg^−1^ N, 7.0 g kg^−1^ P and 7.1 g kg^−1^ K (from 2002 to 2005). For crop straw, the average nutrient content was 8.5 g kg^−1^ N, 2.0 g kg^−1^ P and 17.5 g kg^−1^ K. The plots were ploughed once to a depth of 20 cm using a mouldboard plough. Typically, wheat was irrigated twice at sowing and stem elongation stages, each event with 75 mm of water; whereas maize was irrigated once with 75 mm of water at sowing. Organic manure for NPKM and maize straw for NPKS were applied as base fertilizers before sowing wheat. All P and K fertilizers and 50–70% of N fertilizer were applied as base fertilizers during the sowing period, with the remaining N fertilizer applied as a top-dressing in small holes near the plants at stem elongation stage. During the maize season, soil disturbance was minimal and the fertilizers were applied in small holes near the plants. For wheat, each year the application rates of N, P and K were 165 kg, 36 kg and 68 kg ha^−1^ year^−1^, respectively^45^. For maize, the application rates of N, P and K were 187.5 kg, 41 kg and 78 kg ha^−1^ year^−1^, respectively^45^. The amount of fertilizer applied for each crop under the different treatments is shown in Supplementary Table S3. Crop residues were incorporated into soils in NPKS but were removed from the fields in the other treatments.

### Soil sampling and aggregate fractionation

Soil samples were collected from each plot at a depth of 0–20 cm on June 9, 2013 at wheat harvest stage. The sampling date was selected after the wheat harvest because we mainly paid attentions on the responses of soil nematode community within soil aggregates to inorganic or organic fertilizers throughout the whole growing season rather than different growing stages. Additionally, we observed that the correlation between soil physicochemical properties and soil biological communities were stronger in the wheat season than in the maize season in our previous studies^46^. In each plot, three undisturbed soil blocks (each 10 cm in length, 10 cm in width and 20 cm in depth) were collected randomly with a shovel after removing the surface residue; the samples were composited as a single replicate after sieving according to different aggregate size fractions. A total of 12 soil samples (36 blocks) were collected. Fresh samples were placed in a plastic box and stored at 4 °C until processing and analyses.

Soil aggregate fractions were separated using the dry sieving method described by Gartzia-Bengoetxea *et al.*^47^. Dry sieving was used because in comparison with drying-rewetting cycles, external mechanical stresses are likely the primary reason for aggregate breakdown in agricultural ecosystems^47^. Additionally, dry sieving causes less disruption to the habitats of soil microorganisms than wet-sieving^48^. Before sieving, fresh soil samples were maintained at 4 °C until soils reached a gravimetric water content of approximately 100 g H_2_O kg^−1^ so that finer sieves could be used^16^. Although dry sieving might lead to a loss in soil moisture, the difference in soil moisture between before and after sieving was not significant; thus, the loss of soil moisture was assumed to be identical in all soil samples. After the removal of visible plant residues and stones, the three undisturbed soil blocks in a plot were all sieved through a 7 mm screen by hand and then mixed together uniformly. Soil aggregates were separated by placing 100 g of cool-dried subsamples (<7 mm) onto a nest of sieves mounted on a Retsch AS200 Control (Retsch Technology, Düsseldorf, Germany), which were mechanically shaken (amplitude 1.5 mm) for 2 min to divide the soils into the following four aggregate size classes: >2 mm (large macroaggregates), 1–2 mm (macroaggregates), 0.25–1 mm (small macroaggregates) and <0.25 mm (microaggregates and silt and clay fractions)^49^. The separation procedure was run many times until the three undisturbed soil blocks were all separated into different fractions. The fractionated samples for each aggregate-size class from each sieving were combined to make composite samples for determinations of soil organic carbon, total nitrogen, soil pH, and nematode and microbial communities (PLFAs).

### Soil physicochemical analyses

The mean weight diameter (MWD) was used as an index to reflect the structure of bulk soil. MWD was calculated using the following equation: MWD = ∑X_*j*_W_*j*_, where X_*j*_ is the mean diameter of the size classes and W_*j*_ is the percentage of sample weight on the sieve^50^.

Soil moisture (SM) was measured gravimetrically. Soil organic carbon (SOC) and total nitrogen (TN) within aggregates were measured using a TOC analyser (Multi C/N 3000; Analytik Jena, Germany). Before SOC analysis, the soil samples were fumigated with HCl vapour to remove carbonates^51^. To study the characteristics of carbon and nitrogen pools within aggregates, the element enrichment factors (E) were calculated for each aggregate fraction. The enrichment factors of SOC and TN were calculated using the following equations: E_SOC_ = (g C kg^−1^ fraction)/(g C kg^−1^ soil) and E_TN_ = (g N kg^−1^ fraction)/(g N kg^−1^ soil)^38^,^52^. Bulk soil SOC and TN in these experimental plots are reported in Zhang *et al.*^46^. Soil pH was determined with a glass electrode in 1:2.5 soil: water solution (w/v)^34^.

### Soil PLFA analysis

Analysis of phospholipid fatty acids (PLFAs) was used to characterize the composition of the soil microbial community, according to the method of Bossio and Scow^53^. Lipids were extracted from 8 g freeze-dried soil using a mixture of chloroform: methanol: citrate buffer (1:2:0.8). Polar lipids were separated from neutral lipids and glycolipids on solid phase extraction columns (Supelco Inc., Bellefonte, PA, USA). Phospholipids were treated with a mild-alkali methanolysis and the produced fatty acid methyl esters were subsequently extracted in hexane and dried under N_2_. Samples were analysed using an Agilent 7890A series Gas Chromatograph equipped with MIDI peak identification software (Version 4.5; MIDI Inc., Newark, DE, USA). Before analysis, samples were dissolved in hexane that contained 19:0 as an internal standard. The following markers were chosen to represent bacterial biomass (i.e., 14:0, i14:0, 15:0, i15:0, a15:0, 16:0, i16:0, 16:1 ω7c, 16:1 ω9c, 17:0, i17:0, a17:0, 17:1 ω8c, cy 17:0, 18:0, 18:1 ω5c, 18:1 ω7c, 18:1 ω9c, and cy 19:0), and 18:2 ω6c represented fungal biomass^46^.

### Nematode faunal composition and ecological indices

Nematodes were extracted from 50 g fresh soil by a modified cotton-wool filter method^54^. Nematode abundance was expressed as individuals per 100 g dry soil. After counting total nematode abundance in each sample, 100 individuals were randomly selected and identified to genus. When total nematodes were fewer than 100 in a sample, all nematodes in that sample were identified. Nematodes were assigned to the following trophic groups according to feeding habits: bacterivores, fungivores, omnivores-predators and plant-parasites^55^. Nematode enrichment (EI) and structure (SI) indexes were calculated according to Ferris *et al.*^33^. Average fresh body mass of each nematode genus was estimated according to http://plpnemweb.ucdavis.edu/nemaplex. To gain insight into the metabolic activity levels of different indicator guilds of nematodes, Ferris^56^ proposed the nematode metabolic footprint, which provides a quantitative component of ecosystem structure and function based on carbon use. The nematode metabolic footprint was calculated as ∑ (N_t_ (0.1(W_t_/m_t_) + 0.273 (W^0.75^)), where W_t_ and m_t_ are the body weight and colonizer-persister (cp) values of genus t^57^, respectively. The enrichment footprint and structure footprint represent the metabolic footprints of lower trophic level nematodes (cp 1–2) and higher trophic level nematodes (cp 3–5), respectively. The functional metabolic footprint of nematodes was calculated according to the following formula: (enrichment footprint × structure footprint)/2 with complex μg^2^ units^46^,^56^.

### Statistical analyses

Soil microbial biomass, nematode abundance and nematode functional metabolic footprint were ln(x + 1) transformed before statistical analyses. To test the main influences and interactions of fertilization and aggregate size effects, general linear model analysis of variance designed for split plot was performed with fertilization and aggregate size as fixed factors and replicates as a random factor. Individual comparisons among the treatments for the identical aggregate size fraction were based on Tukey’s honestly significant difference (Tukey HSD) test. Linear correlations were performed to analyse the relationships between nematodes and soil properties within soil aggregates. All statistical analyses were performed using the SPSS 16.0 statistical software package (SPSS Inc., Chicago, IL, USA). Differences at P < 0.05 were statistically significant. Principal components analysis (PCA) was applied to explore the soil nematode community composition according to the relative abundances within aggregates under different fertilization treatments. Redundancy analysis (RDA) was performed to explore the multivariate relationships between nematodes and soil physicochemical properties within aggregates using CANOCO software^58^. A Monte Carlo permutation test (499 permutations) was used to test the significance of the canonical axes.

## Additional Information

**How to cite this article**: Zhang, Z. *et al.* Effect of long-term combined application of organic and inorganic fertilizers on soil nematode communities within aggregates. *Sci. Rep.* **6**, 31118; doi: 10.1038/srep31118 (2016).

## Supplementary Material

Supplementary Information

## Figures and Tables

**Table 1:** The soil aggregate distribution and mean weight diameter (MWD) under different fertilization treatments in the wheat season (mean with standard error in parentheses, n = 3).

Fertilization treatments	Aggregate proportion in size class (%)				MWD (mm)
	>2 mm	1–2 mm	0.25–1 mm	<0.25 mm	
CK	28.62 (1.28)C	20.69 (1.03)AB	40.35 (2.22)A	10.34 (2.42)A	2.01 (0.07)C
NPK	35.79 (0.68)B	19.80 (0.43)B	35.38 (1.21)A	9.03 (0.79)A	2.32 (0.03)B
NPKM	34.15 (0.87)B	22.79 (0.70)AB	39.06 (1.34)A	4.00 (0.90)B	2.30 (0.05)B
NPKS	40.55 (0.90)A	23.53 (0.74)A	33.57 (1.30)A	2.35 (0.98)B	2.59 (0.03)A

Different capital letters represent significant differences among fertilization treatments within the same aggregate fraction in the wheat season, as determined by Tukey’s honestly significant difference test, P < 0.05.

**Table 2:** Soil moisture, SOC, TN, C/N ratios, pH, E_SOC_ and E_TN_ within different aggregate fractions under different fertilization treatments (mean with standard error in parentheses, n = 3).

Soil properties	Fertilization treatments	Aggregate size				Effects		
		>2 mm	1–2 mm	0.25–1 mm	<0.25 mm	F	As	F*As
Soil moisture	CK	7.49(0.71)B	7.51(0.82)B	6.70(0.50)B	4.71(0.18)A	**	**	**
(%)	NPK	9.43(1.20)B	8.39(0.76)B	8.24(0.36)B	5.59(0.23)A			
	NPKM	11.05(2.05)B	9.29(1.80)B	9.91(1.76)B	6.74(0.71)A			
	NPKS	13.32(0.34)A	13.39(1.08)A	11.70(0.85)A	6.46 (0.65)A			
		ab	a	b	c			
SOC	CK	5.95(0.30)B	6.26(0.49)B	5.33(0.02)B	3.99(0.18)C	**	**	*
(g kg^−1^)	NPK	6.31(0.28)B	6.67(0.27)B	5.98(0.30)B	5.00(0.05)B			
	NPKM	9.03(0.41)A	10.20(0.34)A	9.24(0.55)A	7.28(0.17)A			
	NPKS	7.79(0.21)A	8.64(0.53)A	8.50(0.17)A	7.45(0.12)A			
		b	a	b	c			
TN	CK	0.49(0.03)B	0.47(0.02)B	0.43(0.00)B	0.36(0.02)C	**	**	ns
(g kg^−1^)	NPK	0.58(0.02)B	0.60(0.02)B	0.56(0.05)B	0.52(0.01)B			
	NPKM	0.89(0.05)A	0.92(0.03)A	0.91(0.03)A	0.76(0.01)A			
	NPKS	0.81(0.05)A	0.87(0.05)A	0.87(0.02)A	0.82(0.02)A			
		a	a	a	b			
C/N	CK	12.28(0.18)A	12.85(0.54)A	12.29(0.06)A	11.10(0.25)A	**	**	ns
	NPK	10.86(0.20)AB	11.05(0.22)B	10.67(0.36)B	9.70(0.22)B			
	NPKM	10.16(0.15)B	11.13(0.17)B	10.17(0.25)B	9.53(0.07)B			
	NPKS	9.65(0.63)B	9.90(0.38)B	9.77(0.05)B	9.13(0.08)B			
		ab	a	b	c			
pH	CK	8.76(0.06)A	8.76(0.01)A	8.71(0.04)A	8.66(0.04)A	**	**	ns
	NPK	8.38(0.02)B	8.40(0.02)B	8.42(0.02)B	8.32(0.03)B			
	NPKM	8.48(0.02)B	8.46(0.04)B	8.43(0.01)B	8.37(0.04)B			
	NPKS	8.43(0.07)B	8.39(0.04)B	8.44(0.05)B	8.28(0.05)B			
		a	a	a	b			
E_SOC_	CK	1.12(0.06)A	1.18(0.10)A	1.00(0.00)A	0.75(0.04)A	ns	**	**
	NPK	0.89(0.05)B	0.94(0.01)A	0.85(0.02)B	0.71(0.03)A			
	NPKM	0.92(0.03)B	1.04(0.03)A	0.94(0.04)AB	0.74(0.02)A			
	NPKS	0.86(0.04)B	0.96(0.08)A	0.94(0.05)AB	0.83(0.03)A			
		b	a	b	c			
E_TN_	CK	1.00(0.07)A	0.96(0.04)A	0.89(0.01)B	0.74(0.05)B	ns	**	*
	NPK	0.84(0.04)A	0.87(0.02)A	0.82(0.04)B	0.75(0.02)B			
	NPKM	0.92(0.04)A	0.95(0.02)A	0.94(0.02)B	0.79(0.02)B			
	NPKS	0.93(0.08)A	1.00(0.07)A	0.99(0.03)A	0.93(0.03)A			
		a	a	a	b			

SOC, soil organic carbon; TN, total nitrogen; C/N, C/N ratio; Enrichment factors of soil organic carbon (E_SOC_) and total nitrogen (E_TN_); F, fertilization; As, aggregate size. **P < 0.01; *P < 0.05; ns, no significant difference. Different capital letters (ABC) represent significant differences among fertilization treatments within the same aggregate fraction and different lowercase letter (abc) indicate significant differences among aggregate fractions in the wheat season, as determined by Tukey’s honestly significant difference test, P < 0.05.

**Table 3:** Soil microbial biomass within aggregates in different fertilization treatments (mean with standard error in parentheses, n = 3).

Microbialgroups	Fertilizationtreatments	Aggregate size				Effects		
		>2 mm	1–2 mm	0.25–1 mm	<0.25 mm	F	As	F*As
Total PLFA	CK	17.42 (0.95)D	17.39 (2.00)C	16.57 (2.49)C	14.89 (0.71)C	**	**	**
(nmol g^−1^)	NPK	25.40 (1.18)C	27.49 (3.10)B	22.26 (1.85)C	23.31 (2.52)B			
	NPKM	40.70 (3.16)A	41.70 (1.45)A	30.17 (1.19)B	34.84 (1.23)A			
	NPKS	35.01 (1.48)B	38.10 (4.08)A	40.64 (5.72)A	37.84 (1.72)A			
		ab	a	b	b			
Bacteria	CK	11.71 (0.37)D	11.60 (0.80)C	11.21 (0.95)C	10.15 (0.25)C	**	**	**
(nmol g^−1^)	NPK	17.23 (0.44)C	18.51 (1.26)B	15.08 (0.87)C	16.33 (1.08)B			
	NPKM	27.89 (1.18)A	28.66 (0.50)A	20.40 (0.74)B	24.47 (0.38)A			
	NPKS	23.59 (0.66)B	25.88 (1.68)A	27.66 (2.21)A	26.38 (0.55)A			
		ab	a	b	ab			
Fungi	CK	0.33 (0.02)B	0.32 (0.03)C	0.30 (0.04)B	0.25 (0.01)C	**	ns	**
(nmol g^−1^)	NPK	0.38 (0.02)B	0.44 (0.07)BC	0.33 (0.02)B	0.34 (0.03)C			
	NPKM	0.66 (0.03)A	0.68 (0.03)AB	0.43 (0.05)B	0.58 (0.01)B			
	NPKS	0.62 (0.04)A	0.70 (0.06)A	0.76 (0.09)A	0.75 (0.03)A			
		ab	a	b	ab			

F, fertilization; As, aggregate size. **P < 0.01; *P < 0.05; ns, no significant difference. Different capital letters (ABC) represent significant differences among fertilization treatments within the same aggregate fraction and different lowercase letter (ab) indicate significant differences among aggregate fractions in the wheat season, as determined by Tukey’s honestly significant difference test, P < 0.05.

**Table 4:** Nematode ecological indices within different aggregate fractions under different fertilization treatments (mean with standard error in parentheses, n = 3).

Ecological indices	Fertilization treatments	Aggregate size				Effects		
		>2 mm	1–2 mm	0.25–1 mm	<0.25 mm	F	As	F*As
EI	CK	35.37(1.57)A	35.48(2.16)A	35.55(0.10)A	20.00(0.01)B	**	ns	**
	NPK	14.84(0.32)B	13.25(1.47)C	12.83(4.35)B	18.56(3.61)B			
	NPKM	24.31(2.46)A	22.90(1.68)B	41.05(0.61)A	23.75(0.72)B			
	NPKS	37.74(6.15)A	43.61(5.39)A	62.19(3.40)A	75.19(6.28)A			
		a	a	a	a			
SI	CK	61.81(1.74)A	62.86(2.86)A	45.98(2.66)AB	43.00(4.04)AB	*	*	**
	NPK	43.78(0.97)AB	50.02(2.04)AB	43.36(5.29)B	41.37(6.55)AB			
	NPKM	51.29(6.26)AB	40.05(5.79)BC	54.71(1.47)AB	26.11(4.58)B			
	NPKS	40.29(5.99)B	33.14(0.66)C	60.73(1.13)A	63.57(4.54)A			
		ab	ab	a	b			

EI, enrichment index; SI structure index; F, fertilization; As, aggregate size. **P < 0.01; *P < 0.05; ns, no significant difference. Different capital letters (ABC) represent significant differences among fertilization treatments within the same aggregate fraction and different lowercase letter (abc) indicate significant differences among aggregate fractions in the wheat season, as determined by Tukey’s honestly significant difference test, P < 0.05.

## References

[b1] Gruzdeva L. I., Matveeva E. M. & Kovalenko T. E. Changes in soil nematode communities under the impact of fertilizers. Eurasian Soil Sci. 40, 681–693 (2007).

[b2] Sarkar S., Singh S. R. & Singh R. P. The effect of organic and inorganic fertilizers on soil physical condition and the productivity of a rice-lentil cropping sequence in India. J. Agric. Sci. 140, 419–425 (2003).

[b3] Talgre L., Lauringson E., Roostalu H., Astover A. & Makke A. Green manure as a nutrient source for succeeding crops. Plant Soil Environ. 58, 275–281 (2012).

[b4] Wang F., Tong Y. A., Zhang J. S., Gao P. C. & Coffie J. N. Effects of various organic materials on soil aggregate stability and soil microbiological properties on the Loess Plateau of China. Plant Soil Environ. 4, 162–168 (2013).

[b5] Zhang H., Ding W., He X., Yu H. & Fan J. Influence of 20-year organic and inorganic fertilization on organic carbon accumulation and microbial community structure of aggregates in an intensively cultivated sandy loam soil. PLoS ONE 9, e92733 (2014). 24667543 10.1371/journal.pone.0092733 PMC3965464

[b6] Gentile R., Vanlauwe B., Chivenge P. & Six J. Interactive effects from combining fertilizer and organic residue inputs on nitrogen transformations. Soil Biol. Biochem. 40, 2375–2384 (2008).

[b7] Chivenge P., Bernard V. & Johan S. Does the combined application of organic and mineral nutrient sources influence maize productivity? A meta-analysis. Plant Soil 342, 1–30 (2011).

[b8] Tisdall J. M. & Oades J. M. Organic matter and water-stable aggregates in soils. Eur. J. Soil. Sci. 62, 141–163 (1982).

[b9] Mikha M. M. & Rice C. W. Tillage and manure effects on soil and aggregate associated carbon and nitrogen. Soil Sci. Soc. Am. J. 68, 809–816 (2004).

[b10] Young I. M., Crawford J. W., Nunan N., Otten W. & Spiers A. Microbial distribution in soils: physics and scaling. Adv. Agron. 100, 81–121 (2008).

[b11] Kravchenko A. N. *et al.* Intra-aggregate pore structure influences phylogenetic composition of bacterial community in macroaggregates. Soil Sci. Soc. Am. J. 78, 1924–1939 (2014).

[b12] Ferris H. & Matute M. Structural and functional succession in the nematode fauna of a soil food web. Appl. Soil Ecol. 23, 93–110 (2003).

[b13] Ferris H. & Bongers T. Nematode indicators of organic enrichment. J. Nematol. 38, 3–12 (2006). 19259424 PMC2586436

[b14] Quénéhervé P. & Chotte J. L. Distribution of nematodes in vertisol aggregates under a permanent pasture in Martinique. Appl. Soil Ecol. 4, 193–200 (1996).

[b15] Briar S. S. *et al.* The distribution of nematodes and soil microbial communities across soil aggregate fractions and farm management systems. Soil Biol. Biochem. 43, 905–914 (2011).

[b16] Zhang S. X., Li Q., Lü Y., Zhang X. P. & Liang W. J. Contributions of soil biota to C sequestration varied with aggregate fractions under different tillage systems. Soil Biol. Biochem. 62, 147–156 (2013).

[b17] Ettema C. H. & Wardle D. A. Spatial soil ecology. Trends Ecol. Evol. 17, 177–183 (2002).

[b18] Jiang Y. J., Sun B., Jin C. & Wang F. Soil aggregate stratification of nematodes and microbial communities affects the metabolic quotient in an acid soil. Soil Biol. Biochem. 60, 1–9 (2013).

[b19] Mele P. M. & Crowley D. E. Application of self-organizing maps for assessing soil biological quality. Agr. Ecosyst. Environ. 126, 139–152 (2008).

[b20] Zhu H. *et al.* Improving fertility and productivity of a highly-weathered upland soil in subtropical China by incorporating rice straw. Plant Soil 331, 427–437 (2010).

[b21] Zhang P., Wei T., Jia Z. K., Han Q. F. & Ren X. L. Soil aggregate and crop yield changes with different rates of straw incorporation in semiarid areas of northwest China. Geoderma 230–231, 41–49 (2014).

[b22] Roldán A., Salinas-García J. R., Alguacil M. M. & Caravaca F. Soil sustainability indicators following conservation tillage practices under subtropical maize and bean crops. Soil Till. Res. 93, 273–282 (2007).

[b23] Wang K. H., McSorley R., Marshall A. & Gallaher R. N. Influence of organic Crotalaria juncea hay and ammonium nitrate fertilizers on soil nematode communities. Appl. Soil Ecol. 31, 186–198 (2006).

[b24] Antil R. S., Gerzabek M. H., Haberhauer G. & Eder G. Long-term effects of cropped vs. fallow and fertilizer amendments on soil organic matter II. Nitrogen. J. Plant Nutr. Soil Sci. 168, 212–218 (2005).

[b25] Yu H. Y. *et al.* Accumulation of organic C components in soil and aggregates. Sci. Rep-UK 5, 715–719 (2015). 10.1038/srep13804 PMC4566104 26358660

[b26] Xie H. T. *et al.* Long-term manure amendments reduced soil aggregate stability via redistribution of the glomalin-related soil protein in macroaggregates. Sci. Rep-UK 5, 14687 (2015). 10.1038/srep14687 PMC4589770 26423355

[b27] Lichter K. *et al.* Aggregation and C and N contents of soil organic matter fractions in a permanent raised-bed planting system in the highlands of central Mexico. Plant Soil 305, 237–252 (2008).

[b28] Tripathi R. *et al.* Soil aggregation and distribution of carbon and nitrogen in different fractions after 41 years long-term fertilizer experiment in tropical rice–rice system. Geoderma 213, 280–286 (2014).

[b29] Briar S. S., Grewal P. S., Somasekhar N., Stinner D. & Miller S. A. Soil nematode community, organic matter, microbial biomass and nitrogen dynamics infield plots transitioning from conventional to organic management. Appl. Soil Ecol. 37, 256–266 (2007).

[b30] Hati K. M., Mandal K. G., Misra A. K., Ghosh P. K. & Bandyopadhyay K. K. Effect of inorganic fertilizer and farmyard manure on soil physical properties, root distribution, and water-use efficiency of soybean in Vertisols of central India. Bioresource Technol. 97, 2182–2188 (2006). 10.1016/j.biortech.2005.09.033 16289791

[b31] Gryndler M. *et al.* Organic and mineral fertilization, respectively, increase and decrease the development of external mycelium of arbuscular mycorrhizal fungi in a long-term field experiment. Mycorrhiza 16, 159–166 (2006). 16341895 10.1007/s00572-005-0027-4

[b32] Stock S. P. & Goodrich-Blair H. Chapter XII–Nematode parasites, pathogens and associates of insects and invertebrates of economic importance. Manual of Techniques in Invertebrate Pathology 16, 373–426 (2012).

[b33] Ferris H., Bongers T. & de Goede R. G. M. A framework for soil food web diagnostics: extension of the nematode faunal analysis concept. Appl. Soil Ecol. 18, 13–29 (2001).

[b34] Liang W. J., Lou Y. L., Li Q., Zhong S., Zhang X. K. & Wang J. K. Nematode faunal response to long-term application of nitrogen fertilizer and organic manure in Northeast China. Soil Biol. Biochem. 41, 883–890 (2009).

[b35] Ciobanu M., Popovici I., Zhao J. & Stoica I. A. Patterns of relative magnitudes of soil energy channels and their relationships with environmental factors in different ecosystems in romania. Sci. Rep-UK 5, 17606 (2015). 10.1038/srep17606 PMC4664958 26620189

[b36] Zhang Z. Y., Zhang X. K., Jhao J. S., Zhang X. P. & Liang W. J. Tillage and rotation effects on community composition and metabolic footprints of soil nematodes in a black soil. Eur. J. Soil Biol. 66, 40–48 (2015).

[b37] Chen X. F., Li Z. P., Liu M., Jiang C. Y. & Che Y. P. Microbial community and functional diversity associated with different aggregate fractions of a paddy soil fertilized with organic manure and/or NPK fertilizer for 20 years. J. Soil Sediment 15, 292–301 (2015).

[b38] Chen Y. *et al.* Carbon and nitrogen pools in different aggregates of a chinese mollisol as influenced by long-term fertilization. J. Soil Sediment 10, 1018–1026 (2010).

[b39] Jiang Y. J., Jin C. & Sun B. Soil aggregate stratification of nematodes and ammonia oxidizers affects nitrification in an acid soil. Environ. Microbiol. 16, 3083–3094 (2014). 24245556 10.1111/1462-2920.12339

[b40] Jiang Y. J. *et al.* Aggregate-related changes in network patterns of nematodes and ammonia oxidizers in an acidic soil. Soil Biol. Biochem. 88, 101–109 (2015).

[b41] Li Q. *et al.* Nitrogen addition and warming independently influence the belowground micro-food web in a temperate steppe. PLoS ONE 8, e60441 (2013). 23544140 10.1371/journal.pone.0060441 PMC3609780

[b42] Wei C. *et al.* Nitrogen addition regulates soil nematode community composition through ammonium suppression. PLoS ONE 7, 1601–1620 (2012). 10.1371/journal.pone.0043384 PMC3432042 22952671

[b43] Lal M. & Jauhari R. K. Effect of soil pH on the population of migratory nematode Pratylenchus penetrans (Cobb, 1917) (Nematoda: Hoplolamidae) on tea plantations in Doon Valley. J. Exp. Zool. India 10, 469–471 (2007).

[b44] Wang Y. C. *et al.* Crop productivity and nutrient use efficiency as affected by long-term fertilisation in North China Plain. Nutr. Cycl. Agroecosys. 86, 105–119 (2010).

[b45] Huang S., Ma Y., Bao D., Guo D. & Zhang S. Manures behave similar to superphosphate in phosphorus accumulation in long-term field soils. Int. J. Plant Prod. 5, 135–146 (2011).

[b46] Zhang Z. Y., Zhang X. K., Xu M. G., Zhang S. Q., Huang S. M. & Liang W. J. Responses of soil micro-food web to long-term fertilization in a wheat–maize rotation system. Appl. Soil Ecol. 98, 56–64 (2016).

[b47] Gartzia-Bengoetxea N., González-Arias A., Merino A. & de Arano I. M. Soil organic matter in soil physical fractions in adjacent semi-natural and cultivated stands in temperate Atlantic forests. Soil Biol. Biochem. 41, 1674–1683 (2009).

[b48] Schutter M. E. & Dick R. P. Microbial community profiles and activities among aggregates of winter fallow and cover-cropped soil. Soil Sci. Soc. Am. J. 66, 142–153 (2002).

[b49] Zhang S. X. *et al.* Effects of conservation tillage on soil aggregation and aggregate binding agents in black soil of Northeast China. Soil Till. Res. 124, 196–202 (2012).

[b50] Kemper W. D. & Rosenau R. C. Aggregate stability and size distribution. Soil Sci. Soc. Am. J. 9, 425–442 (1986).

[b51] Harris D., Horwath W. R. & van Kessel C. Acid fumigation of soils to remove carbonates prior to total organic carbon or carbon-13 isotopic analysis. Soil Sci. Soc. Am. J. 65, 1853–1856 (2001).

[b52] Amelung W. *et al.* Carbon, nitrogen, and sulfur pools in particle-size fractions as influenced by climate. Soil Sci. Soc. Am. J. 62, 172–181 (1998).

[b53] Bossio D. A., Scow K. M., Gunapala N. & Graham K. J. Determinants of soil microbial communities: effects of agricultural management, season, and soil type on phospholipid fatty acid profiles. Microb. Ecol. 36, 1–12 (1998). 9622559 10.1007/s002489900087

[b54] Townshend J. L. A modification and evaluation of the apparatus for the Oostenbrink direct cottonwool filter extraction method. Nematologica 9, 106–110 (1963).

[b55] Yeates G. W., Bongers T., de Goede R. G. M., Freckman D. W. & Georgieva S. S. Feeding habits in soil nematode families and generae an outline for soil ecologists. J. Nematol. 25, 315–331 (1993). 19279775 PMC2619405

[b56] Ferris H. Form and function: metabolic footprints of nematodes in the soil food web. Eur. J. Soil Biol. 46, 97–104 (2010).

[b57] Bongers T. The maturity index: an ecological measure of environmental disturbance based on nematode species composition. Oecologia 83, 14–19 (1990). 10.1007/BF00324627 28313236

[b58] ter Braak, C. J. F. CANOCO-A FORTRAN Program for Canonical Community Ordination by (partial) (detrended) (canonical) Correspondence Analysis, Principal Components Analysis and Redundancy Analysis (version2.1), Technical Report LWA-88-02. Agricultural Mathematics Group, Wageningen University, 1988.

