# Supplementary material for: Effect of long-term combined application of organic and inorganic fertilizers on soil nematode communities within aggregates
Source: Sci Rep. 2016 Aug 9;6:31118. doi: 10.1038/srep31118 (PMC4977470; doi:10.1038/srep31118)
Supplement: Supplementary Information [file srep31118-s1.doc]

**Effect of long-term combined application of organic and inorganic fertilizers on soil nematode communities within aggregates**

**Zhiyong Zhang ^1,2^, Xiaoke Zhang ^1^,** **Md. Mahamood ^1^, Shuiqing Zhang ^3^, Shaomin Huang ^3^ & Wenju Liang ^1,*^**

^1^ Liaoning Key Laboratory of Soil Environmental Quality and Agro-product Safety, Institute of Applied Ecology, Chinese Academy of Sciences, Shenyang 110016, China

^2^ College of Land and Environment, Shenyang Agricultural University, Shenyang 110161, China

^3^ Institute of Plant Nutrition and Environmental Resources Science, Henan Academy of Agricultural Sciences, Zhengzhou 450002, China

**^*^**Corresponding author: Prof. Wenju Liang

Institute of Applied Ecology

Chinese Academy of Sciences

P. O. Box 417, Shenyang 110016, China

Tel.: +86-24-83970359

Fax: +86-24-83970300

E-mail address: [liangwj@iae.ac.cn](mailto:liangwj@iae.ac.cn)

**Table S1** Proportional contribution (%) of various nematode genera to the nematode assemblage within soil aggregate fractions under different fertilization treatments in the wheat season (mean, n=3).

| Aggregate  Size | Wheat | Bacterivores | | | | | | Fungivores | | | | | | Omnivores-predators | | | | Plant-parasites | | | | | |
| --- | --- | --- | --- | --- | --- | --- | --- | --- | --- | --- | --- | --- | --- | --- | --- | --- | --- | --- | --- | --- | --- | --- | --- |
|  |  | *Mes* | *Cep* | *Euc* | *Acr* | *Pri* | *Ala* | *Fil* | *Dit* | *Par* | *Aph* | *Dip* | *Tyl* | *Tho* | *Eud* | *Epi* | *Dor* | *Bol* | *Bas* | *Geo* | *Hel* | *Pru* | *Pro* |
| > 2 mm | CK | 0.00 | 6.47 | 2.25 | 10.97 | 0.96 | 1.60 | 19.97 | 0.00 | 3.86 | 0.00 | 8.08 | 5.75 | 1.29 | 0.98 | 0.00 | 2.94 | 0.96 | 0.96 | 23.90 | 5.19 | 0.32 | 3.55 |
|  | NPK | 0.00 | 3.57 | 6.63 | 12.48 | 1.67 | 0.00 | 3.54 | 0.00 | 0.00 | 1.93 | 0.00 | 1.26 | 4.21 | 0.98 | 0.33 | 2.31 | 0.33 | 0.00 | 28.89 | 0.64 | 5.90 | 25.33 |
|  | NPKM | 0.30 | 6.08 | 2.82 | 9.98 | 0.96 | 0.67 | 0.93 | 0.30 | 0.30 | 1.86 | 0.00 | 0.00 | 3.19 | 0.63 | 0.00 | 0.93 | 0.00 | 0.00 | 30.52 | 1.00 | 11.27 | 28.26 |
|  | NPKS | 2.22 | 9.16 | 3.59 | 11.03 | 1.32 | 0.66 | 5.77 | 0.00 | 0.00 | 1.00 | 0.00 | 0.97 | 2.93 | 0.33 | 0.00 | 0.00 | 0.00 | 0.00 | 19.09 | 0.33 | 12.02 | 29.56 |
| 1-2 mm | CK | 0.67 | 5.33 | 5.33 | 10.00 | 1.33 | 2.67 | 16.00 | 0.67 | 2.33 | 1.00 | 7.33 | 4.67 | 2.33 | 0.00 | 0.00 | 2.67 | 1.33 | 1.00 | 20.67 | 5.33 | 0.33 | 9.00 |
|  | NPK | 0.00 | 6.20 | 2.62 | 14.68 | 1.97 | 0.66 | 1.97 | 0.00 | 0.00 | 0.66 | 0.00 | 0.33 | 2.29 | 0.33 | 0.00 | 1.62 | 0.00 | 0.00 | 26.33 | 0.66 | 12.49 | 27.20 |
|  | NPKM | 0.33 | 3.65 | 0.99 | 9.97 | 0.67 | 0.66 | 1.00 | 0.33 | 0.33 | 1.33 | 0.00 | 0.33 | 1.33 | 0.00 | 0.00 | 0.33 | 0.00 | 0.00 | 29.90 | 1.00 | 12.98 | 34.87 |
|  | NPKS | 2.32 | 5.27 | 0.67 | 10.24 | 0.33 | 0.67 | 4.27 | 0.00 | 0.00 | 0.67 | 0.00 | 0.33 | 0.98 | 0.00 | 0.00 | 0.00 | 0.00 | 0.00 | 19.32 | 0.99 | 14.83 | 39.11 |
| 0.25-1 mm | CK | 1.67 | 5.31 | 2.66 | 8.64 | 1.99 | 0.99 | 13.27 | 0.99 | 1.00 | 4.00 | 6.97 | 1.00 | 2.99 | 0.33 | 0.00 | 0.33 | 5.66 | 0.33 | 17.94 | 12.28 | 0.00 | 11.65 |
|  | NPK | 0.00 | 2.65 | 2.32 | 8.94 | 0.67 | 0.00 | 1.33 | 0.33 | 0.33 | 0.65 | 0.00 | 0.00 | 1.99 | 0.33 | 0.00 | 0.65 | 0.33 | 0.00 | 19.55 | 0.33 | 14.90 | 44.71 |
|  | NPKM | 0.99 | 1.65 | 2.32 | 5.95 | 1.99 | 1.32 | 1.33 | 0.00 | 0.00 | 1.99 | 0.00 | 0.00 | 0.99 | 0.00 | 0.00 | 0.00 | 0.00 | 0.00 | 23.47 | 1.32 | 16.90 | 39.76 |
|  | NPKS | 5.65 | 1.99 | 2.32 | 6.32 | 0.67 | 1.00 | 5.30 | 0.00 | 0.00 | 0.66 | 0.00 | 0.33 | 2.00 | 0.33 | 0.00 | 0.33 | 0.00 | 0.33 | 12.95 | 0.66 | 17.94 | 41.21 |
| < 0.25 mm | CK | 2.22 | 5.04 | 1.28 | 13.24 | 0.00 | 3.50 | 9.48 | 0.00 | 0.00 | 0.00 | 1.19 | 2.56 | 2.22 | 0.00 | 0.00 | 2.22 | 0.00 | 0.00 | 14.65 | 29.02 | 1.19 | 12.18 |
|  | NPK | 0.46 | 9.15 | 2.78 | 9.14 | 0.00 | 1.04 | 1.04 | 0.46 | 0.46 | 1.63 | 0.00 | 0.00 | 2.32 | 0.00 | 0.00 | 0.93 | 0.81 | 0.00 | 19.58 | 0.00 | 12.40 | 37.79 |
|  | NPKM | 0.67 | 5.32 | 3.63 | 10.36 | 0.00 | 0.00 | 1.36 | 0.00 | 0.00 | 0.33 | 0.00 | 0.00 | 3.29 | 0.00 | 0.00 | 0.33 | 0.00 | 0.00 | 17.32 | 4.96 | 11.91 | 40.52 |
|  | NPKS | 16.01 | 7.23 | 2.39 | 5.77 | 0.00 | 0.34 | 2.34 | 0.00 | 0.00 | 1.01 | 0.00 | 1.05 | 1.42 | 0.00 | 0.00 | 1.76 | 0.00 | 0.00 | 13.99 | 0.34 | 10.50 | 35.87 |

*Mesorhabditis, Mes; Cephalobus, Cep; Eucephalobus, Euc; Acrobeloides, Acr; Prismatolaimus, Pri; Alaimus, Ala; Filenchus, Fil; Ditylenchus, Dit; Paraphelenchus, Par; Aphelenchoides, Aph; Diphtherophora, Dip; Tylencholaimus, Tyl; Thonus, Tho; Eudorylaimus, Eud; Epidorylaimus, Epi; Dorydorella, Dor; Boleodorus, Bol; Basiria, Bas; Geocenamus, Geo; Helicotylenchus, Hel; Pratylenchus, Pru; Pratylenchoides, Pro;*

**Table S2** Correlation between nematode genera and soil properties within aggregates in this study.

|  | Nematode genera | Soil moisture | SOC | TN | C/N | pH |
| --- | --- | --- | --- | --- | --- | --- |
| Bacterivores | *Mesorhabditis* | -0.093 | 0.098 | 0.256 | -0.422 | -0.302 |
|  | *Cephalobus* | -0.135 | -0.291 | -0.177 | -0.176 | -0.113 |
|  | *Eucephalobus* | 0.007 | -0.21 | -0.231 | 0.15 | -0.018 |
|  | *Acrobeloides* | -0.113 | -0.381 | -0.464 | 0.375 | 0.233 |
|  | *Prismatolaimus* | 0.208 | 0.115 | -0.058 | 0.444 | 0.214 |
|  | *Alaimus* | -0.276 | -0.385 | -0.492 | .547* | .737** |
| Fungivores | *Filenchus* | -0.218 | -0.45 | -.597* | .776** | .897** |
|  | *Ditylenchus* | -0.246 | -0.297 | -0.458 | .686** | .646** |
|  | *Paraphelenchus* | -0.223 | -0.286 | -0.444 | .701** | .755** |
|  | *Aphelenchoides* | 0.055 | -0.002 | -0.046 | 0.139 | 0.012 |
|  | *Diphtherophora* | -0.322 | -0.404 | -.584* | .837** | .880** |
|  | *Tylencholaimus* | -0.336 | -0.451 | -.579* | .694** | .799** |
| Omnivores | *Thonus* | 0.038 | -0.273 | -0.235 | 0.007 | -0.024 |
| Predators | *Eudorylaimus* | 0.110 | -0.001 | -0.065 | 0.208 | 0.192 |
|  | *Epidorylaimus* | a | a | a | a | a |
|  | *Dorydorella* | -0.474 | -.527* | -.599* | .503* | 0.405 |
| Plant-parasites | *Boleodorus* | -0.305 | -0.38 | -0.465 | .517* | .511* |
|  | *Basiria* | -0.187 | -0.227 | -0.398 | .684** | .709** |
|  | *Geocenamus* | 0.212 | 0.334 | 0.15 | 0.283 | 0.019 |
|  | *Helicotylenchus* | -.527* | -.587* | -.597* | 0.373 | .575* |
|  | *Pratylenchus* | .537* | .655** | .780** | -.773** | -.805** |
|  | *Pratylenchoides* | 0.355 | .516* | .677** | -.815** | -.871** |

SOC, soil organic carbon; TN, soil total nitrogen; C/N, C/N ratio; Significant levels: **P < 0.01; *P < 0.05. a represent the values can not be calculated by statistical analysis.

**Table** **S3** The average gain yield from 1991 to 2005 and the amount of fertilizers applied for each crop under different treatments.

| Treatments | Wheat (kg ha^-1^) | | | Yield (kg ha^-1^) | Maize (kg ha^-1^) | | | Yield (kg ha^-1^) |
| --- | --- | --- | --- | --- | --- | --- | --- | --- |
|  | urea | superphosphate | potassium  sulphate |  | urea | superphosphate | potassium  sulphate |  |
| CK | 0 | 0 | 0 | 1900B | 0 | 0 | 0 | 3100B |
| NPK | 165 | 36 | 68 | 6200A | 187.5 | 41 | 78 | 6300A |
| NPKM | 49.5+115.5* | 36+64* | 68+83* | 5700A | 187.5 | 41 | 78 | 6500A |
| NPKS | 49.5+115.5* | 36+15* | 68+69* | 6000A | 187.5 | 41 | 78 | 7000A |

*****represent the amount of nitrogen, phosphorus, potassium contained in the added manure or crop straw**.** Different capital letters (A and B) represent significant differences among fertilization treatments.
